# Recent Patient Characteristics and Medications at Admission and Discharge in Hospitalized Patients With Heart Failure

**DOI:** 10.14740/jocmr2402w

**Published:** 2015-12-28

**Authors:** Tadaaki Arimura, Shin-ichiro Miura, Natsumi Morito, Yuhei Shiga, Ken Kitajima, Joji Morii, Atsushi Iwata, Kanta Fujimi, Eiji Yahiro, Keijiro Saku

**Affiliations:** a Department of Cardiology, Fukuoka University School of Medicine, Fukuoka, Japan

**Keywords:** Heart failure, Clinical characteristics, β-blocker, Aldosterone antagonist, Tolvaptan

## Abstract

**Background:**

To improve the clinical outcome of heart failure (HF), it is important to evaluate the etiology and comorbidities of HF. We previously reported the baseline clinical characteristics and medications in hospitalized patients with HF in years 2000 - 2002 (group 2000) and 2007 - 2009 (group 2008).

**Methods:**

We conducted a retrospective study of 158 patients who were hospitalized due to HF between 2012 and 2014 (group 2013) in the Department of Cardiology, Fukuoka University Hospital. We analyzed the clinical characteristics and medications at admission and discharge, and compared the findings in group 2013 to those in group 2000 and group 2008.

**Results:**

The major causes of HF were ischemic heart disease, hypertensive cardiomyopathy, valvular heart disease, and dilated cardiomyopathy. The New York Heart Association classification in group 2013 was significantly higher than those in group 2000 and group 2008. There was no difference in the level of brain natriuretic peptide at admission between group 2008 and group 2013. Tolvaptan began to be administered in group 2013. The median dose of furosemide just before the use of tolvaptan was 40 mg/day. At discharge, group 2013 showed higher rates of β-blocker and aldosterone antagonist. There was no difference in the frequency of loop diuretics. The dose of carvedilol at discharge was only 6.2 ± 4.0 mg/day. Antiarrhythmic drugs and β-blocker were used more frequently in HF with reduced ejection fraction (EF) than in HF with preserved EF.

**Conclusions:**

We may be able to improve the clinical outcome of HF by examining the differences in the clinical characteristics and medications at admission and discharge in hospitalized patients with HF.

## Introduction

Heart failure (HF) has a poor prognosis. However, previous studies have shown that several medications can improve the prognosis of HF [1-14]. It is important that patients are provided the optimal medications according to guidelines for HF [15, 16]. Unfortunately, not all patients with HF receive appropriate medical treatment, and there are differences in the compliance with guidelines between hospitals [17]. In addition, the prognosis is known to worsen if the compliance with guidelines is poor [18]. Previous registration studies include ADHERE (characteristics and outcomes of patients hospitalized for heart failure in the United States) [19], ATTEND (acute decompensated heart failure syndromes registry) [20] and EHFSII (EuroHeart Failure Survey II: a survey of hospitalized acute heart failure patients) [21]. Although these studies included patients with similar ages and genders, there were differences in complications and the duration of hospitalization. The use of medications also differed. We may be able to provide appropriate medical treatment by knowing the present patient characteristics and medications in our hospital. We previously reported the baseline clinical characteristics and medications of hospitalized patients with HF in years 2000 - 2002 (group 2000) and 2007 - 2009 (group 2008) [22]. Various clinical trials have recently been performed in the field of HF [23-29], and new medications are now available [30]. Therefore, we analyzed the patient characteristics and medications at admission and discharge, and compared the findings in 2012 - 2014 (group 2013) to those in group 2000 and group 2008.

## Methods

### Study population

We retrospectively examined the records of patients who had been hospitalized with a main disease of HF in group 2013 in the Department of Cardiology, Fukuoka University Hospital. We compared the features in group 2013 with those in group 2000 and group 2008.

The cause of HF was classified as dilated cardiomyopathy (DCM), hypertrophic cardiomyopathy (HCM), dilated phase of hypertrophic cardiomyopathy (D-HCM), arrhythmogenic right ventricular cardiomyopathy (ARVC), congenital heart disease, ischemic heart disease (IHD), hypertensive cardiomyopathy (HTCM), valvular heart disease, arrhythmia, pulmonary hypertension, sarcoidosis, peripartum cardiomyopathy, myocarditis, or unknown. When the causes of HF overlapped, the main cause of HF was assumed based on the patient’s medical history.

### Clinical parameters

The blood pressure and heart rate were determined, and echocardiography was performed at admission. The echocardiographic parameters examined were the left atrial dimension (LAd), left ventricular end diastolic dimension (LVEDd) and LV ejection fraction (LVEF).

Information regarding medications was collected at three time points (at admission, in the hospital and at discharge). Data on the body mass index (BMI), systolic blood pressure (SBP), diastolic blood pressure (DBP), blood levels of brain natriuretic peptide (BNP), amino-terminal pro-brain natriuretic peptide (NT-proBNP), creatinine (Cr), estimated glomerular filtration rate (eGFR), creatinine clearance (CCr), uric acid (UA), sodium (Na), potassium (K), hemoglobin (Hb), C-reactive protein (CRP), total cholesterol (TC), triglyceride (TG), high-density lipoprotein-cholesterol (HDL-c) and low-density lipoprotein-cholesterol (LDL-c) were also collected at admission. HFrEF (HF with reduced EF) was defined as EF equal to or less than 40%. HFpEF (HF with preserved EF) was defined as EF equal to or more than 50%, and borderline was defined as EF 41-49% [15].

### Statistical analysis

The statistical analysis was performed using Ekuseru-Tokei 2012 software (Social Survey Research Information Co., Ltd) at Fukuoka University Hospital. All data are shown as the mean ± standard deviation, median (minimum - maximum) or median (interquartile range (IQR)). Categorical and continuous variables were compared between the groups by Chi-square analysis and Mann-Whitney U test, respectively. A value of P < 0.05 was considered significant.

## Results

### Patient characteristics at admission

Table 1 shows the patient characteristics at admission in group 2013. The average age was 74 years. Percentage (%) male, hypertension, diabetes mellitus and chronic kidney disease were 54%, 59%, 34% and 75%, respectively. The New York Heart Association classification (NYHA) in group 2013 (3.5 ± 0.7) was significantly higher than those in group 2000 and group 2008 (group 2000 = 2.7 ± 0.8 vs. group 2013 (P < 0.05); group 2008 = 2.6 ± 0.7 vs. group 2013 (P < 0.05)) [22]. BNP and NT-proBNP were measured in 126 (80%) and 110 (70%) patients, respectively, and the averages of these values were 126 pg/mL and 7,569 pg/mL, respectively. There was no difference in %HFrEF (41%) or %HFpEF (42%).

**Table 1:** Patient Characteristics at Admission

	Group 2013 (n = 158)
Male, n (%)	86 (54)
Age	74 ± 13
Height, m	1.58 ± 0.10
Weight, kg	59.2 ± 14.4
NYHA classification	3.5 ± 0.7
Hospitalized days, days	21 ± 13
HTN, n (%)	94 (59)
DM, n (%)	53 (34)
DL, n (%)	80 (51)
CKD, n (%)	118 (75)
Anemia, n (%)	117 (74)
Smoking, current, n (%)	21 (13)
Smoking, former, n (%)	36 (23)
PM, n (%)	16 (10)
ICD, n (%)	18 (11)
CRT, n (%)	6 (4)
SBP, mm Hg	137 ± 31
DBP, mm Hg	78 ± 18
HR, /min	86 ± 24
Biochemical parameters	
BNP, pg/mL	824 ± 702
NT-proBNP, pg/mL	7,569 ± 8,993
Cr, mg/dL	1.3 ± 0.7
eGFR, mL/min/1.73 m^2^	45 ± 21
CCr, mL/min	47 ± 31
UA, mg/dL	6.9 ± 2.2
Na, mEq/L	140 ± 4
K, mEq/L	4.2 ± 0.6
Hb, g/dL	11.4 ± 2.4
CRP, mg/dL	2.41 ± 4.57
TC, mg/dL	150 ± 38
TG, mg/dL	86 ± 35
HDL-C, mg/dL	39 ± 12
LDL-C, mg/dL	89 ± 30
Echocardiographic parameters	
LAd, mm	47.0 ± 8.9
LVEDd, mm	52.5 ± 10.5
LVEF, %	44.7 ± 17.4

NYHA: New York Heart Association; HTN: hypertension; DM: diabetes mellitus; DL: dyslipidemia; CKD: chronic kidney disease; PMI: pacemaker implantation; ICD: implantable cardioverter defibrillator; CRT: cardiac resynchronization therapy; SBP: systolic blood pressure; DBP: diastolic blood pressure; HR: heart rate; BNP: brain natriuretic peptide; NT-proBNP: amino-terminal pro-BNP; Cr: creatinine; eGFR: estimated glomerular filtration rate; CCr: creatinine clearance; UA: uric acid; Na: sodium; K: potassium; Hb: hemoglobin; CRP: C-reactive protein; TC: total cholesterol; TG: triglyceride; HDL-c: high-density lipoprotein-cholesterol; LDL-c: low-density lipoprotein-cholesterol; LAd: left atrial dimension; LVEDd: left ventricular end diastolic dimension; LVEF: left ventricular ejection fraction.

The average duration of hospitalization in group 2013 (21 days) was shorter than those in group 2000 (29 days) and group 2008 (24 days) [22]. Group 2013 (4%) showed a higher use of cardiac resynchronization therapy (CRT) than group 2000 (0%) and group 2008 (2%) [22]. BNP and NT-proBNP were measured in 126 (80%) and 110 (70%) patients, respectively. There was no difference in the level of BNP at admission between group 2008 (853 pg/mL) [22] and group 2013 (824 pg/mL). Seven patients (4.4%) died during hospitalization. The causes of death were infections (n = 3, 1.9%), HF (n = 3, 1.9%) and arrhythmia (n = 1, 0.6%).

### Major causes of HF

The major causes of HF in group 2013 were IHD (30%), HTCM (20%), valvular heart disease (22%) and DCM (10%) (Table 2). These results were similar to those in group 2000 and group 2008 (IHD: group 2000, 39% and group 2008, 37%; HTCM: 15% and 17%; valvular heart disease: 16% and 10%; DCM: 9% and 12%, respectively) [22]. The % valvular heart disease in group 2013 was significantly higher than that in group 2008 (10%).

**Table 2:** Major Courses of HF

DCM, n (%)	16 (10)
HCM, n (%)	4 (3)
D-HCM, n (%)	1 (1)
ARVC, n (%)	1 (1)
Congenital, n (%)	1 (1)
IHD, n (%)	47 (30)
HTCM, n (%)	32 (20)
Valvular heart disease, n (%)	35 (22)
Arrhythmia, n (%)	5 (3)
PH, n (%)	1 (1)
Sarcoidosis, n (%)	1 (1)
Peripartum cardiomyopathy, n (%)	1 (1)
Myocarditis, n (%)	1 (1)
Unknown, n (%)	12 (8)

HF: heart failure; DCM: dilated cardiomyopathy; HCM: hypertrophic cardiomyopathy; D-HCM: dilated phase of HCM; ARVC: arrhythmogenic right ventricular cardiomyopathy; IHD: ischemic heart disease; HTCM: hypertensive cardiomyopathy; PH: pulmonary hypertension.

### Medications in the acute phase of HF

**Figure 1:**
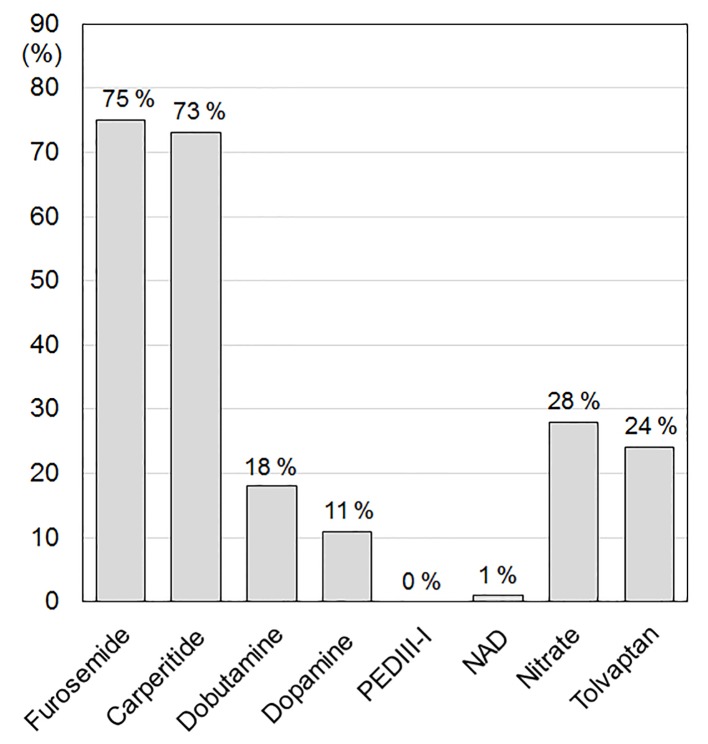
Medications in the acute phase of HF. PDEIII-I: phosphodiesterase inhibitor; NAD: noradrenaline.

The % furosemide, carperitide, dobutamine, dopamine, phosphodiesterase 3 inhibitor (PDEIII-I), nitrate and tolvaptan in group 2013 were 75%, 73%, 18%, 11%, 0%, 28% and 24%, respectively (Fig. 1). During the acute phase of HF, group 2013 showed significantly higher rates of furosemide (vs. group 2000 (54%), P < 0.05) and carperitide (vs. group 2000 (20%), P < 0.05) [22]. The % dopamine and PDEIII-I in group 2013 were significantly decreased compared with those in group 2000 (dopamine, 24%; PEDIII-I, 4%) and group 2008 (dopamine, 20%; PEDIII-I, 8%) [22].

### Dose of furosemide just before the use of tolvaptan

**Figure 2:**
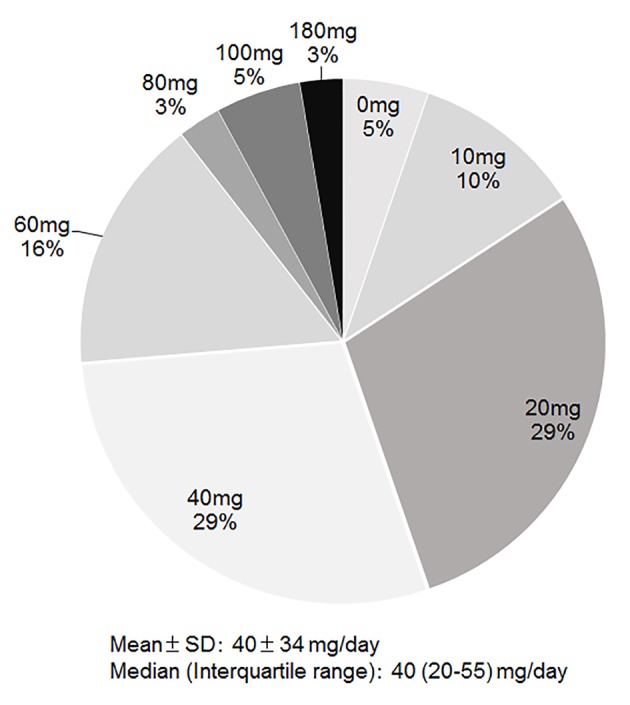
Dose of furosemide just before the use of tolvaptan.

Tolvaptan began to be used in 2010, and its % use in group 2013 was 24%. The dose of tolvaptan was 1.875 mg/day (n = 1), 3.75 mg/day (n = 12), 7.5 mg/day (n = 23) or 15 mg/day (n = 2). As shown in Figure 2, the median dose of furosemide just before the use of tolvaptan was 40 mg/day (IQR 20 - 55 mg/day), when we converted azosemide 30 mg and torasemide 4 mg to furosemide 20 mg. Seventy-nine percent of patients were given tolvaptan with carperitide, and 21% of patients were only given tolvaptan.

### Medications at discharge

**Figure 3:**
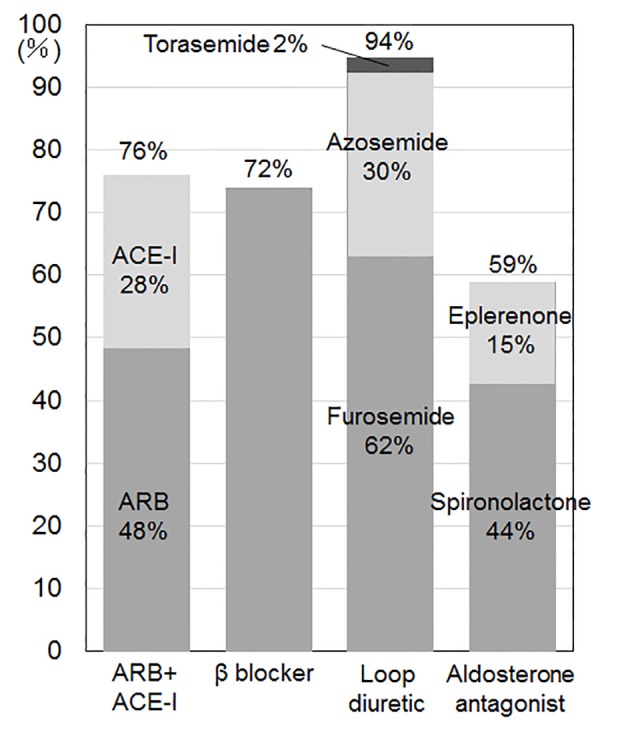
Medications at discharge. ARB: angiotensin receptor blocker; ACE-I: angiotensin converting enzyme inhibitor.

The %ARB + ACE-I, β-blocker, loop diuretic and aldosterone antagonist at discharge were 76%, 72%, 94% and 59%, respectively (Fig. 3). In comparison with group 2000 and group 2008 at discharge [22], group 2013 at discharge showed higher %β-blocker (vs. group 2000 (24%), P < 0.05; vs. group 2008 (48%), P < 0.05) and aldosterone antagonist (vs. group 2000 (43%), P < 0.05). The %ARB and ACE-I were 51% and 25% (group 2000), 56% and 8% (group 2008) and 45% and 28% (group 2013), respectively [22]. There were no differences in the use of loop diuretics. Although furosemide was the only loop diuretic in group 2000 (94%) and group 2008 (95%), azosemide (2%) and torasemide (30%) were also used in group 2013 [22].

### Average dose of carvedilol at discharge

**Figure 4:**
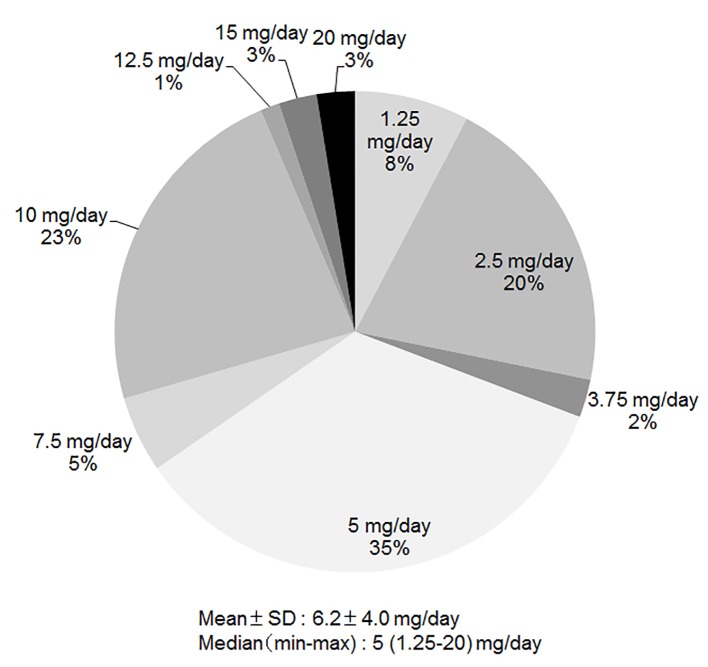
Average dose of carvedilol at discharge.

As shown in Fig. 4, the average dose of carvedilol at discharge was only 6.2 ± 4.0 mg/day. Doses of 2.5 mg/day, 5 mg/day and 10 mg/day were used in 21%, 35% and 23%, respectively.

### Medications in patients with HFrEF, borderline and HFpEF according to LVEF

**Figure 5:**
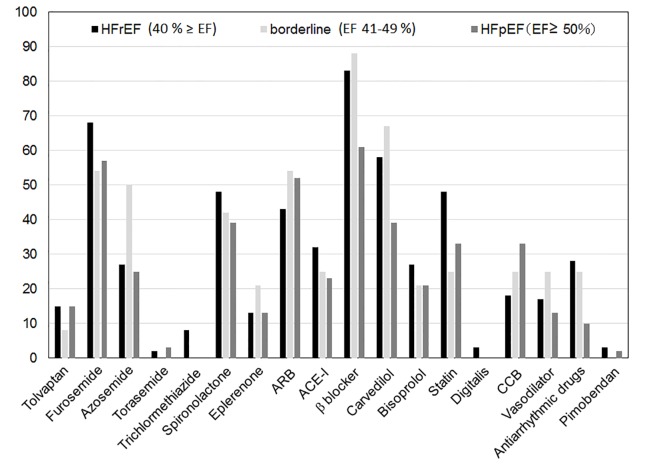
Medications in patients with HFrEF, borderline and HFpEF according to LVEF. HFrEF: heart failure with reduced ejection fraction; HFpEF: heart failure with preserved ejection fraction; ARB: angiotensin receptor blocker; ACE-I: angiotensin converting enzyme inhibitor; CCB: calcium-channel blocker.

Figure 5 shows the medications used in patients with HFrEF, borderline and HFpEF according to LVEF. Antiarrhythmic drugs and β-blocker were used more often in HFrEF (28% and 83%, respectively) than in HFpEF (10% and 61%, respectively) (P < 0.05). In particular, carvedilol was used more often in HFrEF (58%) than in HFpEF (39%) (P < 0.05).

### Cardiac rehabilitation

In our hospital, cardiac rehabilitation was started in 2011. The induction rate of cardiac rehabilitation during hospitalization in group 2013 was 85% (n = 135). On the other hand, only 5% (n = 8) of patients received cardiac rehabilitation as outpatients.

## Discussion

In this study, the major causes of HF were IHD, HTCM, valvular heart disease and DCM. Group 2013 at discharge showed higher rates of β-blocker and aldosterone antagonist than group 2000, whereas the dose of carvedilol at discharge was only 6.2 ± 4.0 mg/day. There were no differences in the use of ACE-I or ARB between group 2000 and group 2013. Tolvaptan became available for use in group 2013, and was administered in 24% of patients. In addition, antiarrhythmic drugs and β-blocker were used more often in HFrEF than in HFpEF.

In recent studies in the United States and Europe, including ADHERE [19], OPTIMIZE-HF [31] and EHFS II [21], the major causes of HF were IHD (about 30-40%), HTCM (about 20%), and valvular heart disease (about 20%). The causes of HF in registry studies in Japan, including JCARE-CARD [32], CHART [33] and ATTEND [20], were similar to those in our group 2013. Nonetheless, the patients in our study showed various causes of HF, including unknown causes. The etiology of HF needs to be evaluated more precisely.

Previous studies have shown that β-blocker improved the clinical outcome of patients with HFrEF [10-13]. Furthermore, other clinical studies showed that ACE-I [1-3], ARB [4-7] and aldosterone antagonist [8, 9, 34] improved the survival rate of patients with HFrEF. The guidelines for HF refer to these studies and recommend the administration of as many of these medications as possible, based on the condition of patients with HF. β-blocker and aldosterone antagonist were used more often in group 2013 than in group 2000. On the other hand, the dose of carvedilol in this study (6.2 ± 4.0 mg/day) was lower than that in a previous clinical study (45 ± 27 mg/day) [13]. Aldosterone antagonists are recommended in patients with HF (NYHA classification II-IV) who have LVEF of 35% or less, unless contraindicated, to reduce morbidity and mortality [15]. Therefore, we should combine aldosterone antagonists with other effective drugs for HF therapy, although aldosterone antagonists were used more often in group 2013 than in group 2000. In this case, serum K and Cr need to be carefully monitored to avoid hyperkalemia and renal insufficiency.

There was no difference in %ACE-I + ARB between group 2000 and group 2013, whereas there was a difference in %ACE-I or ARB between the groups. The stage of HF in this study was C or D (ACCF/AGA stages of HF). In stage C or D, ACE-I is recommended in patients with HFrEF and current or prior symptoms, unless contraindicated, to reduce morbidity and mortality [15]. ARBs are recommended in patients with HFrEF with current or prior symptoms who do not tolerate ACE-I. In the ADHERE trial [19], a clinical trial that was performed in the United States in 2004, the usage rates of ACE-I and ARB were 41% and 12%. In EHFS II [21], which was performed in Europe in 2004 - 2005, the usage rates of ACE-I and ARB were 71.1% and 10.4%. In JCARE-CARD, which was performed in Japan in 2004 - 2005, the usage rates of ACE-I and ARB were 36.7% and 46.1% [32]. In Japan, ARBs are used more often than in the United States and Europe, probably because of adverse effects (e.g., cough, angioedema, rash and taste disturbances).

Tolvaptan became available in Japan for the treatment of HF in 2010. Gheorghiade et al [35] reported that tolvaptan induced an increase in urine volume, a decrease in body weight and normalization of serum sodium. In the EVEREST study [30], tolvaptan showed short-term effectiveness, but did not improve the long-term prognosis. Tolvaptan has only been used for the treatment of HF in Japan. When sufficient diuresis is not achieved with diuretics such as loop diuretics, tolvaptan is recommended. On the other hand, there is currently no clear evidence regarding whether tolvaptan should be used together with loop diuretics. Based on the results of post-marketing surveillance, the median dose of furosemide just before the administration of tolvaptan was 40 mg/day [36]. The median dose of tolvaptan was also 40 mg/day in this study. Since the IQR is from 20 to 55 mg/day, we should start tolvaptan with furosemide ≥ 40 mg/day.

There is considerable evidence regarding the pharmacological treatment of HFrEF. Beneficial drugs include diuretics, ARB, ACE-I, β-blocker, aldosterone antagonist, hydralazine, isosorbide dinitrate, digoxin, anticoagulants, and omega-3 fatty acids. Based on the data in group 2013, we should increase the dose of carvedilol to improve the clinical outcome of HFrEF. On the other hand, statins, nutritional supplements, hormonal therapies, long-term infusion of a positive inotropic drug, and CCBs appeared to be no effect or harmful in patients with HFrEF. No treatment has been shown to be beneficial in HFpEF. Since β-blockers were not effective in HFpEF, they were not used as much as in HFrEF (HFrEF (83%) vs. HFpEF (61%), P < 0.05)). In addition, we have to consider the higher incidence of HFpEF in Japan (HFpEF (25.4%) vs. HFrEF (58.2%)) [32]. Although %HFpEF was equivalent to %HFrEF in group 2013, the only treatments with evidence class I were diuretics and blood pressure management [15].

Torasemide (30% at discharge) was used in addition to furosemide in group 2013. Furosemide and azosemide are short- and long-acting loop diuretics, respectively. Azosemide, compared with furosemide, reduced the unplanned admission to a hospital for congestive HF [29]. In addition, azosemide suppresses activation of the sympathetic nervous system compared with furosemide in patients with HF [37]. Since a few reports have reported that azosemide is superior to furosemide, further studies will be needed to resolve this issue.

In this study, BNP and NT-proBNP were measured in 80% and 70% of the patients. Although the half-life of BNP in blood is short (20 min), that of NT-proBNP is relatively long (120 min). Since NT-proBNP shows high renal clearance, the correlation between NT-proBNP and BNP is worsened when eGFR is < 30 mL/min/1.73 m^2^. NT-proBNP is more stable than BNP after centrifugal separation and freeze-thaw. Since there is considerable evidence regarding the use of BNP [38-41] and NT-proBNP [42-46] as markers for diagnosis, their measurement is useful for screening and determining the prognosis in HF. It is still unclear whether the measurement of NT-proBNP offers any advantages over the measurement of BNP.

### Conclusions

We may be able to improve the clinical outcome of HF by examining the differences in the clinical characteristics and medications at admission and discharge in hospitalized patients with HF. The present findings suggest that we should increase the doses of β-blocker and administer aldosterone antagonist more frequently in our hospital.

## References

[1] (1987). Effects of enalapril on mortality in severe congestive heart failure. Results of the Cooperative North Scandinavian Enalapril Survival Study (CONSENSUS). The CONSENSUS Trial Study Group. N Engl J Med.

[2] (1991). Effect of enalapril on survival in patients with reduced left ventricular ejection fractions and congestive heart failure. The SOLVD Investigators. N Engl J Med.

[3] Cohn JN, Johnson G, Ziesche S, Cobb F, Francis G, Tristani F, Smith R (1991). A comparison of enalapril with hydralazine-isosorbide dinitrate in the treatment of chronic congestive heart failure. N Engl J Med.

[4] Granger CB, McMurray JJ, Yusuf S, Held P, Michelson EL, Olofsson B, Ostergren J (2003). Effects of candesartan in patients with chronic heart failure and reduced left-ventricular systolic function intolerant to angiotensin-converting-enzyme inhibitors: the CHARM-Alternative trial. Lancet.

[5] Pitt B, Poole-Wilson PA, Segal R, Martinez FA, Dickstein K, Camm AJ, Konstam MA (2000). Effect of losartan compared with captopril on mortality in patients with symptomatic heart failure: randomised trial--the Losartan Heart Failure Survival Study ELITE II. Lancet.

[6] Dickstein K, Kjekshus J (2002). Effects of losartan and captopril on mortality and morbidity in high-risk patients after acute myocardial infarction: the OPTIMAAL randomised trial. Optimal Trial in Myocardial Infarction with Angiotensin II Antagonist Losartan. Lancet.

[7] Cohn JN, Tognoni G (2001). A randomized trial of the angiotensin-receptor blocker valsartan in chronic heart failure. N Engl J Med.

[8] Pitt B, Zannad F, Remme WJ, Cody R, Castaigne A, Perez A, Palensky J (1999). The effect of spironolactone on morbidity and mortality in patients with severe heart failure. Randomized Aldactone Evaluation Study Investigators. N Engl J Med.

[9] Zannad F, McMurray JJ, Krum H, van Veldhuisen DJ, Swedberg K, Shi H, Vincent J (2011). Eplerenone in patients with systolic heart failure and mild symptoms. N Engl J Med.

[10] (1999). Effect of metoprolol CR/XL in chronic heart failure: Metoprolol CR/XL Randomised Intervention Trial in Congestive Heart Failure (MERIT-HF). Lancet.

[11] (1999). The Cardiac Insufficiency Bisoprolol Study II (CIBIS-II): a randomised trial. Lancet.

[12] Bristow MR, Gilbert EM, Abraham WT, Adams KF, Fowler MB, Hershberger RE, Kubo SH (1996). Carvedilol produces dose-related improvements in left ventricular function and survival in subjects with chronic heart failure. MOCHA Investigators. Circulation.

[13] Packer M, Bristow MR, Cohn JN, Colucci WS, Fowler MB, Gilbert EM, Shusterman NH (1996). The effect of carvedilol on morbidity and mortality in patients with chronic heart failure. U.S. Carvedilol Heart Failure Study Group. N Engl J Med.

[14] Packer M, Coats AJ, Fowler MB, Katus HA, Krum H, Mohacsi P, Rouleau JL (2001). Effect of carvedilol on survival in severe chronic heart failure. N Engl J Med.

[15] Yancy CW, Jessup M, Bozkurt B, Butler J, Casey DE, Drazner MH, Fonarow GC (2013). 2013 ACCF/AHA guideline for the management of heart failure: a report of the American College of Cardiology Foundation/American Heart Association Task Force on practice guidelines. Circulation.

[16] McMurray JJ, Adamopoulos S, Anker SD, Auricchio A, Bohm M, Dickstein K, Falk V (2012). ESC Guidelines for the diagnosis and treatment of acute and chronic heart failure 2012: The Task Force for the Diagnosis and Treatment of Acute and Chronic Heart Failure 2012 of the European Society of Cardiology. Developed in collaboration with the Heart Failure Association (HFA) of the ESC. Eur Heart J.

[17] Fonarow GC, Yancy CW, Albert NM, Curtis AB, Stough WG, Gheorghiade M, Heywood JT (2008). Heart failure care in the outpatient cardiology practice setting: findings from IMPROVE HF. Circ Heart Fail.

[18] Peterson ED, Albert NM, Amin A, Patterson JH, Fonarow GC (2008). Implementing critical pathways and a multidisciplinary team approach to cardiovascular disease management. Am J Cardiol.

[19] Sato N, Kajimoto K, Asai K, Mizuno M, Minami Y, Nagashima M, Murai K (2010). Acute decompensated heart failure syndromes (ATTEND) registry. A prospective observational multicenter cohort study: rationale, design, and preliminary data. Am Heart J.

[20] Fonarow GC, Heywood JT, Heidenreich PA, Lopatin M, Yancy CW (2007). Temporal trends in clinical characteristics, treatments, and outcomes for heart failure hospitalizations, 2002 to 2004: findings from Acute Decompensated Heart Failure National Registry (ADHERE). Am Heart J.

[21] Nieminen MS, Brutsaert D, Dickstein K, Drexler H, Follath F, Harjola VP, Hochadel M (2006). EuroHeart Failure Survey II (EHFS II): a survey on hospitalized acute heart failure patients: description of population. Eur Heart J.

[22] Natsumi Morito, Shin-ichiro Miura, Keijiro Saku (2011). Changes in the Baseline Clinical Characteristics of Hospitalized Patients with Congestive Heart Failure. Med Bull Fukuoka Univ.

[23] Pitt B, Pfeffer MA, Assmann SF, Boineau R, Anand IS, Claggett B, Clausell N (2014). Spironolactone for heart failure with preserved ejection fraction. N Engl J Med.

[24] Gheorghiade M, Bohm M, Greene SJ, Fonarow GC, Lewis EF, Zannad F, Solomon SD (2013). Effect of aliskiren on postdischarge mortality and heart failure readmissions among patients hospitalized for heart failure: the ASTRONAUT randomized trial. JAMA.

[25] Hernandez AF, Mi X, Hammill BG, Hammill SC, Heidenreich PA, Masoudi FA, Qualls LG (2012). Associations between aldosterone antagonist therapy and risks of mortality and readmission among patients with heart failure and reduced ejection fraction. JAMA.

[26] Felker GM, Lee KL, Bull DA, Redfield MM, Stevenson LW, Goldsmith SR, LeWinter MM (2011). Diuretic strategies in patients with acute decompensated heart failure. N Engl J Med.

[27] Swedberg K, Komajda M, Bohm M, Borer JS, Ford I, Dubost-Brama A, Lerebours G (2010). Ivabradine and outcomes in chronic heart failure (SHIFT): a randomised placebo-controlled study. Lancet.

[28] Massie BM, Carson PE, McMurray JJ, Komajda M, McKelvie R, Zile MR, Anderson S (2008). Irbesartan in patients with heart failure and preserved ejection fraction. N Engl J Med.

[29] Masuyama T, Tsujino T, Origasa H, Yamamoto K, Akasaka T, Hirano Y, Ohte N (2012). Superiority of long-acting to short-acting loop diuretics in the treatment of congestive heart failure. Circ J.

[30] Konstam MA, Gheorghiade M, Burnett JC, Grinfeld L, Maggioni AP, Swedberg K, Udelson JE (2007). Effects of oral tolvaptan in patients hospitalized for worsening heart failure: the EVEREST Outcome Trial. JAMA.

[31] Gheorghiade M, Abraham WT, Albert NM, Greenberg BH, O'Connor CM, She L, Stough WG (2006). Systolic blood pressure at admission, clinical characteristics, and outcomes in patients hospitalized with acute heart failure. JAMA.

[32] Hamaguchi S, Tsuchihashi-Makaya M, Kinugawa S, Yokota T, Ide T, Takeshita A, Tsutsui H (2009). Chronic kidney disease as an independent risk for long-term adverse outcomes in patients hospitalized with heart failure in Japan. Report from the Japanese Cardiac Registry of Heart Failure in Cardiology (JCARE-CARD). Circ J.

[33] Shiba N, Watanabe J, Shinozaki T, Koseki Y, Sakuma M, Kagaya Y, Shirato K (2004). Analysis of chronic heart failure registry in the Tohoku district: third year follow-up. Circ J.

[34] Pitt B, Remme W, Zannad F, Neaton J, Martinez F, Roniker B, Bittman R (2003). Eplerenone, a selective aldosterone blocker, in patients with left ventricular dysfunction after myocardial infarction. N Engl J Med.

[35] Gheorghiade M, Niazi I, Ouyang J, Czerwiec F, Kambayashi J, Zampino M, Orlandi C (2003). Vasopressin V2-receptor blockade with tolvaptan in patients with chronic heart failure: results from a double-blind, randomized trial. Circulation.

[36] Kinugawa K, Sato N, Inomata T, Shimakawa T, Iwatake N, Mizuguchi K (2014). Efficacy and safety of tolvaptan in heart failure patients with volume overload. Circ J.

[37] Hisatake S, Nanjo S, Fujimoto S, Yamashina S, Yuzawa H, Namiki A, Nakano H (2011). Comparative analysis of the therapeutic effects of long-acting and short-acting loop diuretics in the treatment of chronic heart failure using (123)I-metaiodobenzylguanidine scintigraphy. Eur J Heart Fail.

[38] Nakamura M, Endo H, Nasu M, Arakawa N, Segawa T, Hiramori K (2002). Value of plasma B type natriuretic peptide measurement for heart disease screening in a Japanese population. Heart.

[39] Cowie MR, Struthers AD, Wood DA, Coats AJ, Thompson SG, Poole-Wilson PA, Sutton GC (1997). Value of natriuretic peptides in assessment of patients with possible new heart failure in primary care. Lancet.

[40] Roberts E, Ludman AJ, Dworzynski K, Al-Mohammad A, Cowie MR, McMurray JJ, Mant J (2015). The diagnostic accuracy of the natriuretic peptides in heart failure: systematic review and diagnostic meta-analysis in the acute care setting. BMJ.

[41] Tsuchida K, Tanabe K (2008). Plasma brain natriuretic peptide concentrations and the risk of cardiovascular events and death in general practice. J Cardiol.

[42] Januzzi JL, Camargo CA, Anwaruddin S, Baggish AL, Chen AA, Krauser DG, Tung R (2005). The N-terminal Pro-BNP investigation of dyspnea in the emergency department (PRIDE) study. Am J Cardiol.

[43] Wright SP, Doughty RN, Pearl A, Gamble GD, Whalley GA, Walsh HJ, Gordon G (2003). Plasma amino-terminal pro-brain natriuretic peptide and accuracy of heart-failure diagnosis in primary care: a randomized, controlled trial. J Am Coll Cardiol.

[44] Masson S, Latini R, Anand IS, Vago T, Angelici L, Barlera S, Missov ED (2006). Direct comparison of B-type natriuretic peptide (BNP) and amino-terminal proBNP in a large population of patients with chronic and symptomatic heart failure: the Valsartan Heart Failure (Val-HeFT) data. Clin Chem.

[45] Costello-Boerrigter LC, Boerrigter G, Redfield MM, Rodeheffer RJ, Urban LH, Mahoney DW, Jacobsen SJ (2006). Amino-terminal pro-B-type natriuretic peptide and B-type natriuretic peptide in the general community: determinants and detection of left ventricular dysfunction. J Am Coll Cardiol.

[46] Ng LL, Loke IW, Davies JE, Geeranavar S, Khunti K, Stone MA, Chin DT (2005). Community screening for left ventricular systolic dysfunction using plasma and urinary natriuretic peptides. J Am Coll Cardiol.

